# Real-world vitiligo management in Argentina: Bridging guidelines and practice

**DOI:** 10.1016/j.jdin.2025.12.012

**Published:** 2026-01-05

**Authors:** López Di Noto Ada Laura, Torre Ana Clara, Cura María Julia, Mazzuoccolo Luis Daniel

**Affiliations:** Dermatology Department, Hospital Italiano de Buenos Aires, Buenos Aires, Argentina

**Keywords:** consensus, dermatologist, diagnostic, epidemiology, guideline, treatment, vitiligo

*To the Editor:* Vitiligo affects approximately 1% of the global population and imposes a substantial psychosocial burden. Although several management guidelines exist, data on real-world implementation in Latin America remain scarce. We conducted a survey among Argentine dermatologists to evaluate diagnostic and therapeutic practices for vitiligo and to compare them with international recommendations.^1^, ^2^, ^3^

Between March and May 2024, a 26-item anonymous online survey was distributed by email to 450 board-certified dermatologists across Argentina (Supplementary Information available via Mendeley at https://data.mendeley.com/datasets/z6bmtbz5bs/1). The study adhered to the Strengthening the Reporting of Observational Studies in Epidemiology (STROBE) reporting guidelines. Categorical variables were expressed as absolute and relative frequencies, and statistical analyses were performed using STATA 14.2.

A total of 223 physicians (49.5%) responded. Of these, 104 (47%) had 5 to 15 years of professional experience, 79 (35%) more than 15 years, and 40 (18%) less than 5 years. All respondents practiced in urban regions (>300 inhabitants/km^2^) and treated patients with vitiligo. Their approaches to patient evaluation, diagnostic work-up, and disease severity assessment are summarized in Table I. Topical corticosteroids were the most frequently selected first-line therapy (208, 93%) for cases involving <10% body surface area, while narrowband ultraviolet B phototherapy phototherapy (180, 81%) was preferred for more extensive disease. Second-line choices included phototherapy (149, 66%) and oral corticosteroids (104, 47%), whereas third-line treatments comprised narrowband ultraviolet B phototherapy (86, 38%), camouflage (83, 37%), and systemic immunosuppressants (56, 25%) (Table II). Regarding the perceived impact on quality of life, 109 (48.8%) dermatologists rated it as moderate, 106 (47.5%) as severe, and 8 (3.5%) as mild. Statistical analyses explored associations between physicians’ expertise, geographic region, and management practices, hypothesizing that these variables might influence treatment selection. However, no significant relationships were identified.

**Table I:** Medical interview approach, diagnostic work-up, and severity assessment performed by Argentine dermatologists in vitiligo (*n* = 223)

Variable	*n*	(%)
Medical interview approach		
Including duration, lesions location, associated autoimmune diseases, disease progression, family background, triggers	181	81.17
Laboratory tests		
Thyroid-stimulating hormone	211	94.62
Antithyroid antibodies	187	83.86
Complete blood count	196	87.89
Blood glucose level	171	76.68
25-OH-vitamin D levels	97	43.5
Celiac disease-specific antibodies	86	38.57
Antinuclear antibodies	51	22.87
Wood’s light evaluation	88	39.46
Skin biopsy		
All cases	3	1.35
Doubtful cases	128	57.4
Severity assessment	168	75.34
Physician’s photographs	157	93.45
Patient’s photographs	36	21.43
Dermatology Life Quality Index	19	11.31
Vitiligo Area Scoring Index	11	6.55

**Table II:** Argentine dermatologists’ choices of treatment in vitiligo (*n* = 223)

Treatment	First line		Progressive vitiligo	Second line	Third line	Maintenance
	Less 10% BSA	More 10% BSA				
Total, *n* (%)	223 (100)					125 (56.05)
Topical CS, *n* (%)	208 (93.27)	143 (64.13)	137 (61.43)	54 (24.22)		19 (15.2)
Topical calcineurin inhibitor, *n* (%)	181 (81.17)	136 (60.99)	105 (47.09)	70 (31.39)		79 (63.2)
UVBnb, *n* (%)	68 (30.49)	180 (80.72)	153 (68.61)	149 (66.81)	86 (38.56)	17 (7.62)
PUVA, *n* (%)	7 (3.14)	31 (13.9)	29 (13)	31 (13.9)	29 (13)	
Systemic CS, *n* (%)	6 (2.69)	40 (17.94)	161 (72.20)	104 (46.64)	49 (21.97)	
Psychotherapy, *n* (%)	90 (40.36)	121 (54.26)	110 (49.33)			4 (1.79)
Vitamins, antioxidants, *n* (%)	51 (22.87)	67 (30.4)	52 (23.32)			11 (8.1)
Topical calcipotriol, *n* (%)	31 (13.9)	36 (16.14)	21 (9.42)			
Topical JAKi, *n* (%)	2 (0.9)	4 (1.74)	16 (7.17)			
Excimer laser, *n* (%)	5 (2.24)	4 (1.74)	2 (2.69)			
Camouflage, *n* (%)					83 (37.22)	
Surgical techniques, *n* (%)	5 (2.24)				22 (9.87)	
Nonsteroidal immunosuppressant, *n* (%)				53 (23.77)	56 (25.11)	
Diet, photoprotection, moisturizers, *n* (%)						12 (5.38)
Others, *n* (%)					43 (19.28)	

*BSA*, Body surface area; *CS*, corticosteroids; *JAKi*, Janus kinase inhibitors; *PUVA*, psoralen and ultraviolet light A photochemotherapy; *UVBnb*, narrowband ultraviolet B phototherapy.

Overall, diagnostic approaches and first-line and second-line therapeutic choices among Argentine dermatologists closely followed international guidelines, including those from the United Kingdom, Canada, India, Brazil, and China. Variability observed in third-line management may reflect discrepancies among these guidelines. For example, while the Brazilian consensus discourages permanent depigmentation, British guidelines recommend it.^1^, ^2^, ^3^ Access limitations also shape local practice. Topical Janus kinase inhibitors and traditional Chinese therapies, for instance, are not approved or marketed in Argentina for vitiligo.^4^ Furthermore, half of respondents did not prescribe maintenance therapy despite growing evidence supporting its role in relapse prevention, possibly reflecting a knowledge gap given the recency of these data.^1^ Although nearly all dermatologists acknowledged the moderate-to-severe psychosocial burden of vitiligo, only half recommended psychotherapy as part of initial management. This gap is concerning considering the high prevalence of anxiety and depression among affected individuals.^5^ Limited access to specialized psychological resources may contribute to this finding.

In summary, vitiligo management among Argentine dermatologists largely aligns with international consensus for first-line and second-line therapies. Nonetheless, gaps persist in third-line treatment, maintenance therapy, and psychosocial support. Given the structural inequities affecting health care access across Latin America, these findings underscore the need for regional consensus, improved access to emerging therapies, and stronger integration of psychological care. To our knowledge, this is the first Latin American study to describe real-world vitiligo management and highlight opportunities to optimize patient outcomes.

## Conflicts of interest

Dr Daniel has received honoraria from Abbvie, Pfizer, Sanofi, and Elly Lily for participation in scientific activities. Dr Clara has received honoraria from Abbvie and Sanofi for participation in scientific activities. Dr Julia has received honoraria from Abbvie for participation in scientific activities.
